# Notes on the Morphology and Systematic Position of *Archaeolus* Lin, 1986, from the Jurassic of South China (Coleoptera: Elateroidea)

**DOI:** 10.3390/insects12100876

**Published:** 2021-09-27

**Authors:** Yan-Da Li, Robin Kundrata, Di-Ying Huang, Chen-Yang Cai

**Affiliations:** 1 State Key Laboratory of Palaeobiology and Stratigraphy, Center for Excellence in Life and Palaeoenvironment, Nanjing Institute of Geology and Palaeontology, Chinese Academy of Sciences, Nanjing 210008, China; ydli@nigpas.ac.cn (Y.-D.L.); dyhuang@nigpas.ac.cn (D.-Y.H.); 2 Department of Zoology, Faculty of Science, Palacky University, 77146 Olomouc, Czech Republic; robin.kundrata@upol.cz; 3 School of Earth Sciences, University of Bristol, Life Sciences Building, Tyndall Avenue, Bristol BS8 1TQ, UK

**Keywords:** Elateroidea, Protagrypninae, Throscidae, Mesozoic, *Archaeolus*

## Abstract

**Simple Summary:**

Elateroidea is one of the large superfamilies in the beetle suborder Polyphaga. Many adpression-type elateroid fossils were insufficiently described, which hinders the interpretation of their systematic position. Here, we figure and re-describe an elateroid fossil, *Archaeolus funestus*, from the Jurassic of South China. Our observations support that *Archaeolus* might be a member of the Throscidae family.

**Abstract:**

The morphology of the Jurassic fossil *Archaeolus funestus* Lin, 1986, which was previously placed in the extinct click-beetle subfamily Protagrypninae (Coleoptera: Elateridae), is revised based on a re-examination of the type specimen. The validity of Protagrypninae is discussed and further questioned, partly based on the newly observed characters in *A. funestus*, including the surface sculpture of the mesoventrite. A possible Throscidae affinity of monotypic *Archaeolus* Lin, 1986, as suggested in a recent study, is further critically reviewed.

## 1. Introduction

Elateroidea is one of the large superfamilies in the beetle suborder Polyphaga and contains both hard- and soft-bodied forms. Some hard-bodied elateroid families (i.e., Eucnemidae, Throscidae, Cerophytidae, and Elateridae) share a somewhat uniform appearance, especially due to the presence of a pro-mesothoracic clicking mechanism. Though recent molecular studies have revealed that Eucnemidae, Throscidae, and Cerophytidae are distantly related to the much more diverse and common family Elateridae [1,2,3,4], historically these four families were thought to form a monophyletic clade based on their morphology [5]. In adpression fossils of hard-bodied elateroids, due to limited available characters and often taphonomic artefacts, it is even more difficult to determine their precise systematic position [6,7]. In the present paper, we re-examine the elateroid fossil, *Archaeolus funestus* Lin, 1986, from the Jurassic of South China, and discuss its systematic position.

## 2. Materials and Methods

The holotype of *A. funestus* was collected from the Shiti Formation (Middle Jurassic according to Zhang [8] and Yin et al. [9]) at the Xiwan Coal Mine, Pinggui District, Hezhou City, Guangxi, China [10]. The specimen is deposited in the Nanjing Institute of Geology and Palaeontology (NIGP), Chinese Academy of Sciences, Nanjing, China.

Photographs under incident light were taken with a Zeiss Discovery V20 stereo microscope and stacked in Helicon Focus 7.0.2. Scanning electron microscopic (SEM) images were obtained with a Hitachi SU 3500 scanning electron microscope, operating with an accelerating voltage of 15 kV and a pressure of 70 Pa. Images were further processed in Adobe Photoshop CC to enhance contrast.

## 3. Systematic Palaeontology

Order Coleoptera Linnaeus, 1758

Suborder Polyphaga Emery, 1886

Superfamily Elateroidea Leach, 1815

Family (?) Throscidae Laporte, 1840

 

**Figure 1:**
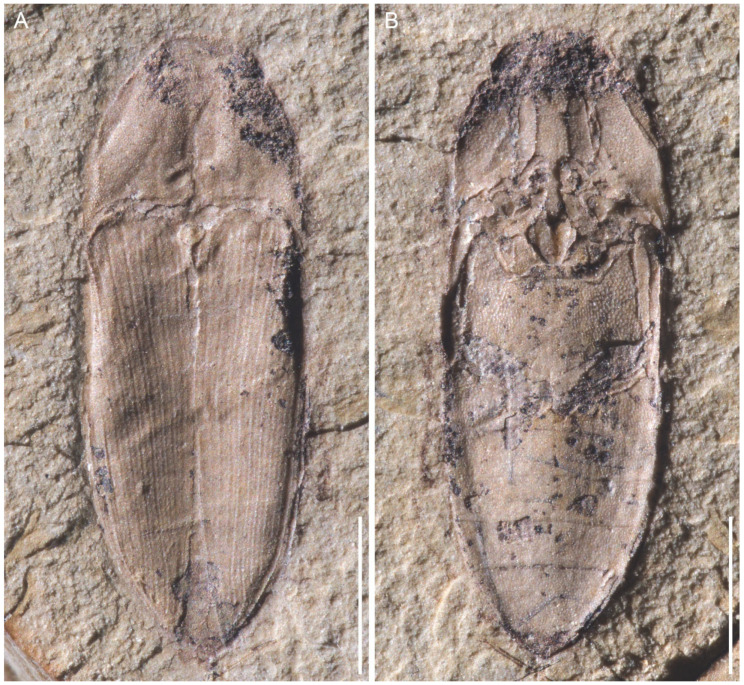
General habitus of *Archaeolus* *funestus* Lin, 1986, holotype, NIGP70071, under incident light. (**A**) Part, NIGP70071a. (**B**) Counterpart, NIGP70071b. Scale bars: 1 mm.

**Figure 2:**
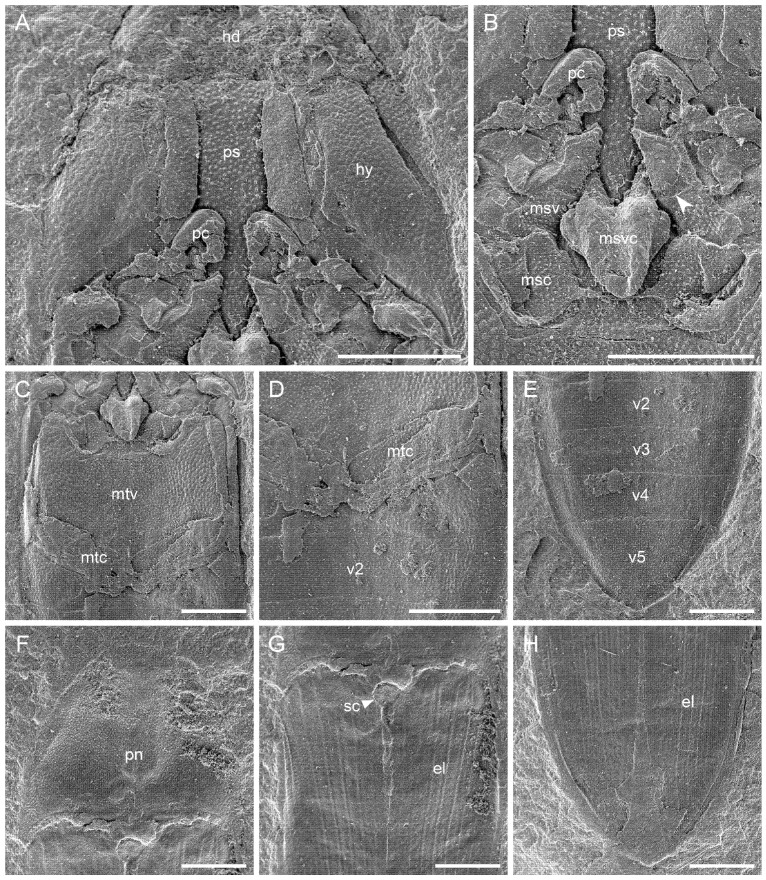
Details of *Archaeolus funestus* Lin, 1986, holotype, NIGP70071, under scanning electron microscopy (SEM). (**A**–**E**) NIGP70071b. (**A**) Head and prothorax. (**B**) Mesothorax, showing the line between mesoventrite body and procoxal rests (arrowhead). (**C**) Metathorax. (**D**) Metacoxae and abdominal base. (**E**) Abdominal apex. (**F**–**H**) NIGP70071a. (**F**) Prothorax. (**G**) Elytral base. (**H**) Elytral apex. Abbreviations: el, elytron; hd, head; hy, hypomeron (pronotum); msc, mesocoxa; msv, mesoventrite; msvc, mesoventral cavity; mtc, metacoxa; mtv, metaventrite; pc, procoxa; pn, pronotum; ps, prosternum; sc, scutellum; v2–5, ventrites 2–5. Scale bars: 400 μm.

*Archaeolus funestus* **Lin, 1986**

(Figure 1 and Figure 2)

 

**Material.** Holotype, sex unknown, NIGP70071 (NIGP).

**Re-description.** Body elongate, length 3.9 mm, width 1.4 mm; surface punctate. Head transverse, not well-preserved. Pronotal disc (Figure 2F) probably less than 1.6 times as wide as long along the middle; sides not sinuate, converging anteriorly; posterior angles strongly acute and produced posteriorly. Elytra (Figure 2G,H) about 2.0 times as long as wide combined, subparallel in anterior half, tapering apically; surface with at least eight punctate striae. Prosternum (Figure 2A) in front of coxae subtrapezoidal, slightly wider basally; prosternal carinae subparallel, diverging near the posterior end; median portion of prosternum between prosternal carinae more than twice as wide as prosternal process (distance between procoxae); prosternal process slender, apically acute (subacute), fitting into mesoventral cavity. Antennal grooves possibly present along pronotosternal suture (Figure 2A). Mesoventrite with distinct procoxal rests (Figure 2B). Metaventrite (Figure 2C) without discrimen; mesotarsal grooves absent. Metacoxal plates (Figure 2D) transverse, with generally parallel sides. Abdomen (Figure 2E) with five ventrites; ventrite 5 about 2.0 times as long as ventrite 4; metatarsal grooves or impressions absent.

**Remarks.** Since there is a relatively wide space between the prosternum and the pronotal hypomeron (at least well shown on one side), we suppose there should be an antennal groove along the pronotosternal suture. The specimen NIGP70071b is generally an impression of the ventral side of the beetle; thus, a groove on the beetle body should appear as a ridge on the impression. However, the pronotosternal suture does not appear as a distinct ridge on that specimen, which we suppose is caused by the damage during fossilisation and (or) fossil preparation.

In previously known throscids, the prosternal carinae, if present, are always continuous with the lateral edges of prosternal process (e.g., [11]). In *Archaeolus*, however, the distance between the prosternal carinae is much wider than the prosternal process, and the carinae are not continuous with the lateral edges of prosternal process, which might be an apomorphy of the genus.

## 4. Discussion

*Archaeolus* was originally included in Elateridae without a specified subfamilial placement [10]. After re-studying the type material, Dong et al. [12] and Ponomarenko et al. [13] placed *Archaeolus* in tribe Protagrypnini within the subfamily Protagrypninae, which are both extinct taxa attributed to Elateridae [14]. However, the validity of Protagrypninae itself is in question. As noticed by previous researchers (e.g., [6,7,15,16]), many species previously classified in Protagrypninae may belong to other extant elaterid subfamilies or even other hard-bodied elateroid families (i.e., Cerophytidae, Eucnemidae, and Throscidae). The extinct tribe Protagrypnini, as defined by Dolin [14,17], is characterised by the presence of a groove along the pronotosternal sutures and the presence of a transverse suture on the mesoventrite, which has been uncritically adopted by some following researchers (e.g., [18,19,20,21,22]). The first character, pronotosternal grooves, can be found in many other extant Elateridae, as well as Throscidae and Eucnemidae. The second diagnostic character, based on our observations, is not a suture at all, and instead, it represents the line between the mesoventrite body and procoxal rests, which is in agreement with the general observations by Kundrata et al. [7]. In the mesoventrite of *Archaeolus*, the portion below the transverse line (mesoventrite body) is developed with small punctures, a type of common surface decoration in elateroids, while the portion above the line is relatively smooth, suggesting it is not usually exposed as the outer surface, supporting an interpretation as procoxal rests (Figure 2B). An additional character indicative of Protagrypninae (including species in Protagrypnini) is the median plate-like structure on the prosternum. A similar structure can also be found in Throscidae, Eucnemidae, and some members of extant Elateridae, and therefore it may have a limited taxonomic value [6,7].

Muona et al. [6], based on the images and the descriptions in Dong et al. [12], transferred *Archaeolus* to the extant family Throscidae. However, our re-examination of the holotype revealed some problems in the illustration and description by Dong et al. [12], which also partly questions the interpretation by Muona et al. [6]. The key character for a placement of *Archaeolus* in Throscidae, as claimed by Muona et al. [6], is the “antennal groove running close to the [pro]notosternal suture and then turning towards the hind corners of the prothorax above the protibial groove”. However, we found no clear evidence supporting the fact that the antennal groove extends beyond the posterior end of the pronotosternal suture and turns towards the hind corners of the prothorax (Figure 2A). Thus, its validity cannot be guaranteed. Dong et al. [12] claimed that *Archaeolus* has distinct antennal clubs, which if true, as noticed by Muona et al. [6], is another typical throscid feature. However, based on our observations, the antennae of *Archaeolus* are essentially not preserved at all (Figure 2A). Metacoxal plates medially extending posteriorly could serve as an additional feature to characterise throscids [23]. Unfortunately, the metacoxae are not well-preserved in the holotype of *A. funestus* (Figure 2D). Therefore, we argue that the systematic position of *Archaeolus* cannot be confidently solved based on the available characters. Nevertheless, considering its general appearance and prosternal structure, we agree with Muona et al. [6] that, at this moment, it is better to place *Archaeolus* to Throscidae rather than retain it in the “wastebin taxon” Protagrypninae.

## References

[1] Kundrata R., Bocakova M., Bocak L. (2014). The comprehensive phylogeny of the superfamily Elateroidea (Coleoptera: Elateriformia). Mol. Phylogenet. Evol..

[2] Zhang S.-Q., Che L.-H., Li Y., Liang D., Pang H., Ślipiński A., Zhang P. (2018). Evolutionary history of Coleoptera revealed by extensive sampling of genes and species. Nat. Comm..

[3] McKenna D.D., Shin S., Ahrens D., Balke M., Beza-Beza C., Clarke D.J., Donath A., Escalona H.E., Friedrich F., Letsch H. (2019). The evolution and genomic basis of beetle diversity. Proc. Natl. Acad. Sci. USA.

[4] Douglas H.B., Kundrata R., Brunke A.J., Escalona H.E., Chapados J.T., Eyres J., Richter R., Savard K., Ślipiński A., McKenna D. (2021). Anchored phylogenomics, evolution and systematics of Elateridae: Are all bioluminescent Elateroidea derived click beetles?. Biology.

[5] Lawrence J.F. (1987). Rhinorhipidae, a new beetle family from Australia, with comments on the phylogeny of the Elateriformia. Invertebr. Taxon..

[6] Muona J., Chang H., Ren D. (2020). The clicking Elateroidea from Chinese Mesozoic deposits (Insecta, Coleoptera). Insects.

[7] Kundrata R., Packova G., Prosvirov A.S., Hoffmannova J. (2021). The fossil record of Elateridae (Coleoptera: Elateroidea): Described species, current problems and future prospects. Insects.

[8] Zhang R.-J., Wang S.-E. (1985). The Central South China Region. Stratigraphy of China (No. 11): The Jurassic System of China.

[9] Yin B.-A., Chen J., Pan L.-Z., Liu R.-T., Zhang J.-Y., Huang H.-W., Liao Q.-K., Yin D.-W., Lu H.-J., Cai N.-H. (1997). Lithostratigraphy of Guangxi Zhuang Autonomous Region.

[10] Lin Q.-B. (1986). Early Mesozoic Fossil Insects from South China.

[11] Li Y.-D., Huang D.-Y., Cai C.-Y. (2021). New genera and species of the family Throscidae (Coleoptera: Elateroidea) in mid-Cretaceous Burmese amber. Insects.

[12] Dong F.-B., Cai C.-Y., Huang D.-Y. (2011). Revision of five Mesozoic beetles from southern China. Acta Palaeontol. Sinica.

[13] Ponomarenko A.G., Yan E.V., Wang B., Zhang H.-C. (2012). Revision of some Early Mesozoic beetles from China. Acta Palaeontol. Sinica.

[14] Dolin V.G., Dolin V.G., Panfilov D.V., Ponomarenko A.G., Pritykina L.N. (1980). Click-beetles (Coleoptera, Elateridae) from Upper Jurrasic of Karatau. Mesozoic Fossil Insects.

[15] Li Y.-D., Tihelka E., Liu Z.-H., Huang D.-Y., Cai C.-Y. (2020). *Muonabuntor* gen. nov., a new genus of false click beetles from mid-Cretaceous Burmese amber (Coleoptera: Elateroidea: Eucnemidae). Palaeoentomology.

[16] Kundrata R., Packova G., Hoffmannova J. (2020). Fossil genera in Elateridae (Insecta, Coleoptera): A Triassic origin and Jurassic diversification. Insects.

[17] Dolin V.G. (1975). A contribution to the systematics of the Mesozoic click-beetles (Coleoptera, Elateridae). Paleontol. J..

[18] Chang H., Zhang F., Ren D. (2008). A new genus and two new species of fossil Elaterids from the Yixian Formation of Western Liaoning, China (Coleoptera: Elateridae). Zootaxa.

[19] Chang H., Zhao Y., Ren D. (2009). New fossil elaterids (Insect: Coleoptera: Polyphaga: Elateridae) from the Middle Jurassic of Inner Mongolia, China. Prog. Nat. Sci..

[20] Dong F., Huang D. (2011). A new elaterid from the Middle Jurassic Daohugou biota (Coleoptera: Elateridae: Protagrypninae). Acta Geol. Sinica.

[21] Dong F.-B., Huang D.-Y. (2009). A new click beetle (Coleoptera: Elateridae) from Middle Jurassic Haifanggou Formation of western Liaoning, China. Acta Palaeontol. Sinica.

[22] Sohn J.-C., Nam G.S., Choi S.-W., Ren D. (2019). New fossils of Elateridae (Insecta, Coleoptera) from Early Cretaceous Jinju Formation (South Korea) with their implications to evolutionary diversity of extinct Protagrypninae. PLoS ONE.

[23] Muona J. (2019). Throscidae (Coleoptera) relationships, with descriptions of new fossil genera and species. Zootaxa.

